# Structured micro‐ultrasonography training improves prostate cancer detection and management decisions

**DOI:** 10.1111/bju.70360

**Published:** 2026-06-18

**Authors:** Maximilian Philip Wessendorf, Maurin Helen Mangold, Gloria Baumann, Nektarios Winter, Johannes Jannik Braun, Jiri Lehmberg, Alexander Studier‐Fischer, Christoph Brochhausen, Maurice Stephan Michel, Sigrid V. Carlsson, Karl‐Friedrich Kowalewski, Caelán Max Haney‐Aubert

**Affiliations:** ^1^ German Cancer Research Centre (Deutsches Krebsforschungszentrum [DKFZ]) Heidelberg Intelligent Systems and Robotics in Urology Heidelberg Germany; ^2^ DKFZ Hector Cancer Institute at the University Medical Centre Mannheim Heidelberg Germany; ^3^ Clinical Epidemiology of Early Cancer Detection German Cancer Research Centre (DKFZ) Heidelberg Germany; ^4^ Department of Urology Mannheim University Medical Centre Mannheim Germany; ^5^ Institute for Pathology Mannheim University Medical Centre Mannheim Germany; ^6^ Division of Urological Cancers, Department of Translational Medicine, Medical Faculty Lund University Lund Sweden

**Keywords:** micro‐ultrasonography, prostate cancer, training, PRI‐MUS, clinically significant prostate cancer, reader study, diagnostic imaging, learning curve

## Abstract

**Objective:**

To evaluate whether a structured 2‐day micro‐ultrasonography (microUS) training curriculum improves diagnostic performance and management decisions for clinically significant prostate cancer (csPCa) detection in novice readers.

**Subjects and Methods:**

A total of 20 participants with limited microUS experience completed the Mannheim Micro‐Ultrasound Curriculum, comprising didactic modules, supervised case interpretation, and 85 reference cases. Pre‐ and post‐training performance was assessed on a fixed, biopsy confirmed test set of 100 cases (50 csPCa confirmed on radical prostatectomy, 50 biopsy negative). Each participant interpreted 10 randomised cases per timepoint, with no case assessed twice per reader and each case being assessed once before and after training in each training session. The primary endpoint was change in cohort‐level accuracy for csPCa detection; secondary endpoints included sensitivity, specificity, predictive values, area under the receiver operating characteristic curve (AUC), Prostate Risk Identification using microUS (PRI‐MUS) performance, and management recommendations. Mixed‐effects models were applied as a robustness analysis.

**Results:**

Training significantly improved pooled cohort level accuracy from 50.5% (95% confidence interval [CI] 43.4–57.6%) to 64.5% (95% CI 57.4–71.1%; *P* = 0.006), sensitivity from 70.0% (95% CI 60.0–78.8%) to 92.0% (95% CI 84.8–96.5%; *P* = 0.001), and negative predictive value from 50.8% (95% CI 37.7–63.9%) to 82.2% (95% CI 67.9–92.0%; *P* = 0.001), whereas specificity did not increase significantly (31.0% vs 37.0%; *P* = 0.46). The AUC for the overall subjective risk score rose from 0.53 (95% CI 0.45–0.61) to 0.70 (95% CI 0.63–0.77; *P* = 0.002). Mixed‐effects modelling confirmed a significant training effect (odds ratio 2.13, 95% CI 1.20–3.79; *P* = 0.01), reproducible across two independent sessions. PRI‐MUS scoring improvements were not statistically significant. Overall decision correctness improved from 67% to 83% (*P* = 0.001), with poor‐quality decisions decreasing from 33% to 17%.

**Conclusion:**

Structured image‐based microUS training significantly improved cohort‐level diagnostic performance and management decisions in novice readers in a controlled reader‐study. These findings support structured training and quality assurance before independent clinical implementation of microUS.

**Abbreviations**
AUC: area under the receiver operating characteristic curve
DKFZ: Deutsches Krebsforschungszentrum (German Cancer Research Centre)
ISUP: International Society of Urological Pathology
microUS: micro‐ultrasonography
mpMRI: multiparametric MRI
(cs)PCa: (clinically significant) prostate cancer
NPV: negative predictive value
PI‐QUAL: Prostate Imaging Quality
PI‐RADS: Prostate Imaging‐Reporting and Data System
PRI‐MUS: Prostate Risk Identification using Micro‐Ultrasonography
ROC: receiver operating characteristic

## Introduction

Prostate cancer (PCa) is the second most commonly diagnosed cancer in men worldwide [1, 2]. Early detection is critical for improving prognosis and survival outcomes [3]. In men with clinical suspicion of PCa, multiparametric MRI (mpMRI) is currently the standard imaging modality [2, 4]. A mpMRI enables sensitive detection of clinically significant PCa (csPCa) [5, 6], but limited availability and high examination costs remain important barriers, particularly in rural areas and lower‐income settings [7, 8, 9].

Micro‐ultrasonography (microUS) is an emerging imaging modality that allows high‐resolution assessment of prostatic tissue and detection of csPCa. Recent evidence suggests that microUS is non‐inferior to mpMRI for the detection of csPCa [10, 11], and its clinical adoption is increasing. This highlights the need to ensure consistent image quality and interpretation as microUS use expands.

In contrast to mpMRI, where structured frameworks such as the Prostate Imaging‐Reporting and Data System (PI‐RADS) for lesion scoring and Prostate Imaging Quality (PI‐QUAL) score for image quality provide established guidance on acquisition, reporting, and minimum quality standards [12, 13, 14, 15], microUS has not yet reached a comparable level of standardisation [16, 17]. Formal requirements for operator training, structured reporting, and quality assurance remain less clearly defined. As a result, diagnostic performance depends heavily on individual operators, and variability in acquisition and interpretation may limit reproducibility and broader clinical implementation.

Several studies have begun to characterise the diagnostic performance and learning curve of microUS, mostly in the context of microUS‐guided targeted biopsy using endpoints such as csPCa detection rates, negative predictive value (NPV), and biopsy quality metrics [16, 18, 19]. A structured training programme reported by Cash et al. [18] showed that biopsy performance improves with increasing microUS experience. However, the proctoring approach proposed by Cash et al. [18] relies on experience gained in live cases, which may lead to suboptimal examinations early in the learning curve.

This study aimed to address this gap by evaluating a structured 2‐day microUS training programme. The primary objective was to determine whether standardised training improves cohort‐level diagnostic performance and management decisions on a biopsy‐confirmed test dataset using randomised pre‐ and post‐training assessments.

## Subjects and Methods

### Ethics and Study Approval

This study was conducted using a prospectively maintained microUS database approved by the institutional ethics committee (2023‐645‐AF 5) at Mannheim University Medical Centre and the Intelligent Systems in Urology Group at the German Cancer Research Centre (Deutsches Krebsforschungszentrum [DKFZ]), Heidelberg, Germany. All patients provided written informed consent for data use. The reader study was approved separately by the institutional ethics committee (vote 2024‐653). The study was registered in the German Clinical Trials Register (DRKS00039253). Patients were recruited between January 2024 and July 2025.

### Participants

A total of 20 participants (10 board‐certified urologists, seven urology residents, and three medical students) completed the full 2‐day training and both evaluation timepoints across two identical training courses with 10 participants each. In all, 11 participants had no prior microUS experience; the others had experience with mpMRI‐fusion biopsies but limited (<40 examinations) microUS diagnostic exposure.

### The MicroUS Examinations

The MicroUS sweeps were acquired using the ExactVu™ 29‐MHz system (Exact Imaging Inc., Markham, Canada) in B‐mode. Examinations were recorded as cine loops at 8–12 frames/s, up to the system's 300‐frame limit and stored in Digital Imaging and Communications in Medicine (DICOM) format. Individual sweeps typically comprised 150–250 frames depending on gland size.

During acquisition, the probe was rotated from the right peripheral zone to the left at constant speed and pressure. Each examination consisted of multiple standardised sweeps, including at least one overview sweep at 50‐mm depth to ensure complete gland visualisation, as well as high‐magnification sweeps of the peripheral zone (base and apex). Additional sweeps with increased gain and probe pressure were performed to optimise visualisation of the transitional zone.

### Training Data

Cases used during training were not selected consecutively but with focus on their didactic value. Examinations included patients with negative prostate biopsy (25), and patients with csPCa (International Society of Urological Pathology [ISUP] Grade Group >1), where microUS was performed either prior to biopsy (40) or radical prostatectomy (20). Biopsy results and, where applicable, whole‐mount radical prostatectomy pathology (30) were discussed for each case. The dataset included examples of benign changes, large tumours of the peripheral and transitional zones, and challenging cases with multifocal lesions or artefacts.

### Course Structure and Content

**Fig. 1:**
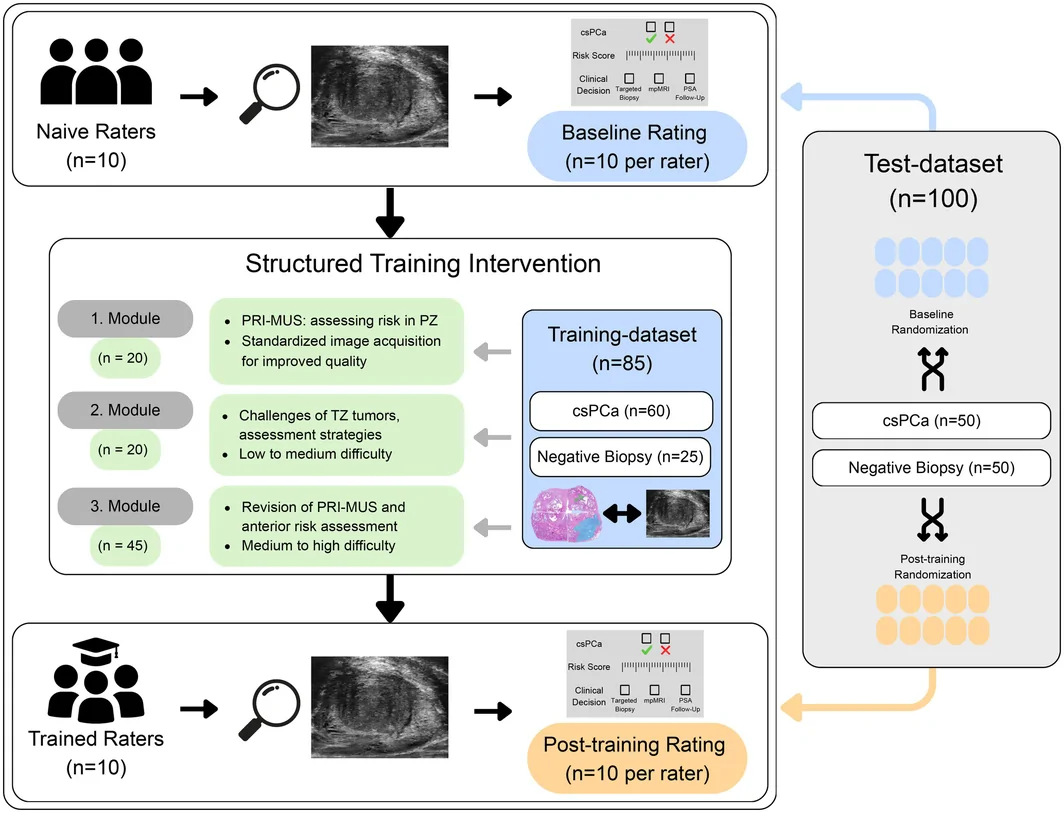
Structure of the Mannheim Micro‐Ultrasound Curriculum and reader‐study design. Overview of the structured 2‐day microUS training curriculum and evaluation workflow. Participants completed baseline assessment on randomised cases from a fixed biopsy‐verified test dataset before undergoing structured didactic training and supervised case review using 85 curated training cases. After training, participants evaluated a second randomised set of cases from the same test dataset, with no reader assessing the same case twice. The curriculum included modules on PRI‐MUS assessment, image acquisition and optimisation, and evaluation of peripheral‐ and transitional‐zone tumours with increasing case complexity. PZ, peripheral zone; TZ, transition zone.

A structured 2‐day microUS training programme was developed (Fig. 1, Video S1) to standardise image acquisition, interpretation, and decision‐making for csPCa independent of mpMRI. The course was delivered by a clinician with extensive microUS experience (>1000 interpreted cases; C.M.H.A.).

Training consisted of a combination of didactic modules, interactive teaching, and supervised case interpretation. Teaching sessions were delivered in person. Cases were presented in Medical Imaging Interaction Toolkit (MITK; DKFZ), using 2–4 standardised microUS cine‐loops. After a brief introduction to prostate anatomy, participants completed a baseline evaluation. Training included didactic sessions on the prostate risk identification using microUS (PRI‐MUS™) protocol as defined by Ghai et al. [20], peripheral‐zone assessment, microUS image optimisation, and tumour detection in the transitional zone, each followed by group‐based case discussions with correlation to histopathology. During training, all 85 curated cases (25 biopsy negative) were reviewed using a standardised workflow of individual interpretation, group discussion, reference standard disclosure, and expert‐led resolution.

### Evaluation

#### Test Data

A fixed test set of 100 cases (50 biopsy positive for csPCa, 50 biopsy negative) was used for evaluation. No test case overlapped with training material.

To construct the test set, the microUS database was consecutively screened for eligibility. Inclusion criteria were mpMRI, biopsy indication at recruitment (PI‐RADS score ≥3, abnormal DRE, or PSA density ≥0.2 ng/mL/mL), and microUS performed before biopsy. Previous biopsy status was not part of case selection and may have been present in both csPCa‐positive and biopsy‐negative patients. Biopsy consisted of ≥12 systematic cores and three to six cores per mpMRI target. For positive cases, only patients with csPCa (ISUP Grade Group >1) on biopsy who subsequently underwent radical prostatectomy with full histopathological evaluation at the Mannheim University Medical Centre were included. In all, 31 examinations were excluded due to very poor image quality, mainly caused by rectal gas artefacts, extensive calcifications, or incomplete prostate visualisation due to suboptimal operator technique or limited patient tolerance. Patient characteristics are presented in Tables 1 and 2.

**Table 1:** Characteristics of the evaluation dataset.

Characteristic	csPCa (*n* = 50)	Negative biopsy (*n* = 50)
PSA level, ng/mL, median (IQR)	7.3 (5.4–10.8)	7.1 (4.9–12.8)
PI‐RADS score category, *n*		
2	1	4
3	3	25
4	29	16
5	17	5
Total	50	50

IQR, interquartile range.

**Table 2:** Pathological characteristics of csPCa‐positive cases (*n* = 50).

Characteristic	*N* (%)
Pathological tumour stage	
pT2a	3 (6)
pT2c	39 (78)
pT3a	4 (8)
pT3b	4 (8)
ISUP Grade Group	
ISUP 2	33 (66)
ISUP 3	15 (30)
ISUP 4	1 (2)
ISUP 5	1 (2)
Tumour location	
Peripheral zone (PZ)	23 (46)
Anterior (TZ + AFS)	10 (20)
Combined anterior/PZ involvement	17 (32)

Percentages may not sum to 100 owing to rounding.

AFS, anterior fibromuscular stroma; pT, pathological tumour stage; PZ, peripheral zone; TZ, transition zone.

#### Assessment Protocol

Each participant interpreted 10 cases from the test dataset before training and 10 different cases after training. Test cases were distributed across participants such that within each cohort of 10 readers all 100 test cases were evaluated exactly once per timepoint. No participant evaluated the same case twice. Within each batch of 10 cases, csPCa prevalence was constrained to three to seven cases using a reproducible randomisation seed. Readers were blinded to clinical and histopathological data and were provided two to four microUS sweeps per case.

For each case, readers completed an on‐line survey indicating the presence or absence of csPCa (yes/no) and assigning three subjective 1–10 risk scores reflecting perceived likelihood of csPCa and diagnostic confidence: an overall risk score, a peripheral‐zone risk score, and a transitional‐zone risk score. The broader 1–10 scale was chosen to allow more nuanced risk stratification while maintaining the simplified three‐tier interpretation framework used during training. During training, scores 1–3 indicated likely benign findings, 4–5 equivocal appearances, and ≥6 suspicious findings. Readers also assigned a PRI‐MUS score (1–5) to the most suspicious lesion and provided a management recommendation (biopsy, mpMRI, or PSA follow‐up).

Decisions were evaluated using two frameworks: a binary correctness model (biopsy for positive cases, follow‐up or mpMRI for negative cases) and a three‐level quality model classifying decisions as good (biopsy for positive cases or follow‐up for negative cases), acceptable (mpMRI), or poor (biopsy for negative cases or follow‐up for positive cases).

### Statistical Analysis

The primary endpoint was the change in pooled accuracy for csPCa detection. Secondary endpoints included sensitivity, specificity, NPV, areas under the receiver operating characteristic (ROC) curves (AUCs) for continuous risk scores, PRI‐MUS‐based performance, and management recommendations. Exploratory analyses assessed risk scores dichotomised at >5, their combined use with PRI‐MUS (PRI‐MUS score >3 or PRI‐MUS score = 3 + overall risk score >5), and zone‐specific scores. Diagnostic performance was analysed separately for pre‐ and post‐training timepoints using pooled reader ratings to estimate group‐level performance. The two training cohorts were pooled for primary analysis because they underwent identical training and evaluated the same test dataset. As cases were interpreted by different readers at each timepoint and no reader assessed the same case twice, analyses were performed at the pooled cohort level rather than as paired within‐reader comparisons. Sensitivity, specificity, positive predictive value, NPV, and accuracy were calculated with exact Clopper–Pearson 95% CIs. Differences in binary outcomes and management recommendations were tested using Fisher's exact tests. ROC curves were generated for continuous risk scores, and AUCs with 95% CIs were estimated using the DeLong method. As a pre‐specified robustness analysis to account for potential clustering by reader and case, mixed‐effects logistic regression with random intercepts for reader and case was performed. This model assessed the robustness of the post‐training effect after accounting for clustering. Reproducibility was assessed by comparing baseline performance and training effects between the two independent sessions. An exploratory model including reader role assessed whether the training effect differed by professional background. Statistical significance was defined as two‐sided *P* ≤ 0.05. As an exploratory educational reader study, sample size was based on participant availability and the number of curated training and test cases.

## Results

### Diagnostic Performance

Overall pooled accuracy increased from 50.5% (95% CI 43.4–57.6%) before training to 64.5% (95% CI 57.4–71.1%) after training (*P* = 0.006). Pooled sensitivity for detecting csPCa increased from 70.0% (95% CI 60.0–78.8%) before training to 92.0% (95% CI 84.8–96.5%) after training (*P* = 0.001). Specificity increased from 31.0% (95% CI 22.1–41.0%) to 37.0% (95% CI 27.6–47.2%), but this change was not statistically significant (*P* = 0.46). Participants labelled 65 cases as negative before training and 79 cases after training with an increase in NPV from 50.8% (95% CI 37.7–63.9%) to 82.2% (95% CI 67.9–92.0%) (*P* = 0.001) (Table 3). Using the predefined cut‐offs (PRI‐MUS score >3 and overall risk score >5), sensitivity increased significantly after training.

**Table 3:** Diagnostic performance metrics before and after training.

Metric	Type	Before training	After training	*P* (for change)
Binary rating (csPCa yes/no)				
Accuracy	Cohort level overall, % (95% CI)	50.5 (43.4–57.6)	64.5 (57.4–71.1)	0.006
Accuracy	Reader level (*n* = 20), %, median (IQR)	50.0 (40.0–60.0)	70.0 (57.5–70.0)	0.015
Sensitivity	Cohort level overall, % (95% CI)	70.0 (60.0–78.8)	92.0 (84.8–96.5)	0.001
Sensitivity	Reader level (*n* = 20), %, median (IQR)	69.0 (60.0–85.0)	100.0 (82.5–100.0)	0.002
Specificity	Cohort level overall, % (95% CI)	31.0 (22.1–41.0)	37.0 (27.6–47.2)	0.46
Specificity	Reader level (*n* = 20), %, median (IQR)	36.7 (19.2–40.0)	40.0 (25.0–50.0)	0.28
NPV	Cohort level overall, % (95% CI)	50.8 (37.7–63.9)	82.2 (67.9–92.0)	0.001
NPV	Reader level (*n* = 18), %, median (IQR)	50.0 (35.0–91.7)	100.0 (66.7–100.0)	0.03
Overall risk score (positive if >5)				
Accuracy	Cohort level overall, % (95% CI)	52.5 (45.3–59.6)	64.5 (57.4–71.1)	0.02
Accuracy	Reader level (*n* = 20), %, median (IQR)	50.0 (40.0–70.0)	70.0 (50.0–72.5)	0.04
Sensitivity	Cohort level overall, % (95% CI)	60.0 (49.7–69.7)	89.0 (81.2–94.4)	0.001
Sensitivity	Reader level (*n* = 20), %, median (IQR)	60.0 (40.0–75.0)	100.0 (80.0–100.0)	0.001
Specificity	Cohort level overall, %, (95% CI)	45.0 (35.0–55.3)	40.0 (30.3–50.3)	0.57
Specificity	Reader level (*n* = 20), %, median (IQR)	40.0 (23.8–60.0)	41.4 (20.0–51.8)	0.64
NPV	Cohort level overall, % (95% CI)	52.9 (41.8–63.9)	78.4 (64.7–88.7)	0.003
NPV	Reader level (*n* = 18), %, median (IQR)	50.0 (42.5–65.6)	100.0 (61.7–100.0)	0.007
PRI‐MUS score (positive if >3)				
Accuracy	Cohort level overall, % (95% CI)	50.0 (42.9–57.1)	52.5 (45.3–59.6)	0.69
Accuracy	Reader level (*n* = 20), %, median (IQR)	50.0 (40.0–60.0)	50.0 (40.0–60.0)	0.65
Sensitivity	Cohort level overall, % (95% CI)	47.0 (36.9–57.2)	67.0 (56.9–76.1)	0.006
Sensitivity	Reader level (*n* = 20), %, median (IQR)	41.4 (25.0–68.8)	63.3 (50.0–87.5)	0.03
Specificity	Cohort level overall, % (95% CI)	53.0 (42.8–63.1)	38.0 (28.5–48.3)	0.05
Specificity	Reader level (*n* = 20), %, median (IQR)	60.0 (40.0–66.7)	40.0 (23.8–50.0)	0.05
NPV	Cohort level overall, % (95% CI)	50.0 (40.1–59.9)	53.5 (41.3–65.5)	0.76
NPV	Reader level (*n* = 17), %, median (IQR)	50.0 (42.9–50.0)	50.0 (33.3–66.7)	0.82
Combined rule (PRI‐MUS score >3 OR PRI‐MUS score = 3 and overall risk score >5)				
Accuracy	Cohort level overall, % (95% CI)	49.5 (42.4–56.6)	57.0 (49.8–64.0)	0.16
Accuracy	Reader level (*n* = 20), %, median (IQR)	50.0 (40.0–60.0)	50.0 (50.0–70.0)	0.19
Sensitivity	Cohort level overall, % (95% CI)	53.0 (42.8–63.1)	84.0 (75.3–90.6)	0.001
Sensitivity	Reader level (*n* = 20), %, median (IQR)	55.0 (40.0–75.0)	100.0 (66.7–100.0)	0.001
Specificity	Cohort level overall, % (95% CI)	46.0 (36.0–56.3)	30.0 (21.2–40.0)	0.03
Specificity	Reader level (*n* = 20), %, median (IQR)	50.0 (31.2–60.0)	25.0 (20.0–40.7)	0.05
NPV	Cohort level overall, % (95% CI)	49.5 (38.9–60.0)	65.2 (49.8–78.6)	0.1
NPV	Reader level (*n* = 16), %, median (IQR)	50.0 (48.2–60.0)	66.7 (47.5–100.0)	0.02

Diagnostic performance is shown for binary csPCa classification, subjective risk score thresholds, PRI‐MUS score thresholds, and the combined decision rule. Cohort‐level analyses are presented with exact 95% CIs, whereas reader‐level analyses are reported as median (IQR) across readers. The *P* values represent comparisons between pre‐ and post‐training performance. Combined rule defined as PRI‐MUS score >3 or PRI‐MUS score = 3 with overall risk score >5.

IQR, interquartile range (quartile 1 to quartile 3).

Mixed‐effects modelling confirmed a significant post‐training effect (odds ratio 2.13, 95% CI 1.20–3.79; *P* = 0.01). Baseline performance did not differ between sessions (*P* = 0.85), and the training effect was similar across sessions (interaction *P* = 0.41) (Table S1 and Fig. S1). An exploratory model including reader role showed no significant interaction between professional background and training effect (*P* = 0.34).

### Risk Scores and ROC Analyses

**Fig. 2:**
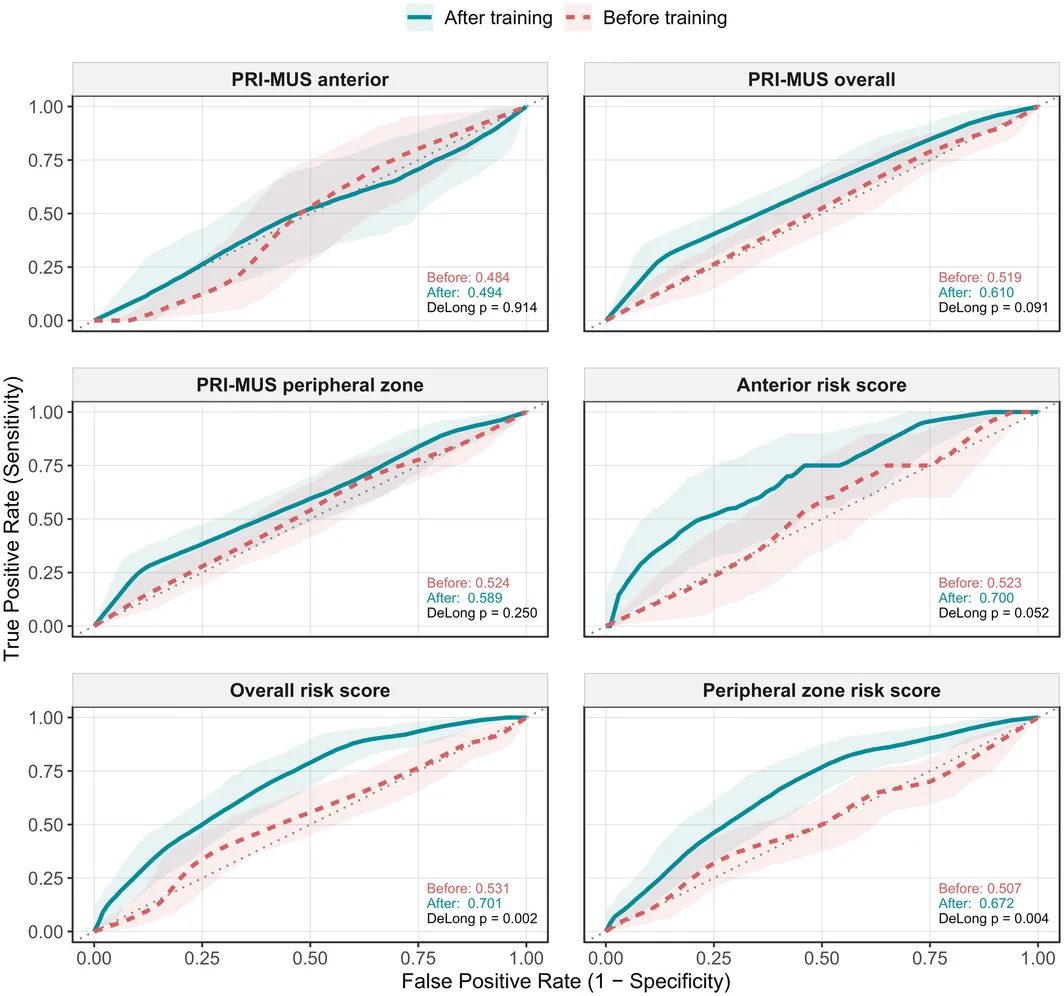
The ROC curves for PRI‐MUS and subjective risk scores before vs after training. The ROC curves for PRI‐MUS and subjective risk scores before and after completion of the Mannheim Micro‐Ultrasound Curriculum. Overall/global metrics were evaluated against csPCa status, peripheral‐zone metrics against peripheral‐zone and mixed peripheral/anterior tumours, and anterior metrics against anterior tumours only. Shaded areas represent bootstrap 95% CIs. The AUC values and *P* values were calculated using the DeLong method.

The overall risk score assigned by the participants achieved an AUC of 0.531 (95% CI 0.451–0.611) before training and 0.701 (95% CI 0.631–0.771) after training (*P* = 0.002). This improvement was caused by a shift towards higher scores in positive cases while the median score for negative cases remained unchanged (Fig. S2). The peripheral zone risk score achieved an AUC of 0.507 (95% CI 0.424–0.590) before training and 0.672 (95% CI 0.597–0.746) after training (*P* = 0.004). Performance of the anterior risk score showed a similar improvement, with an AUC of 0.523 (95% CI 0.395–0.651) before training and 0.700 (95% CI 0.578–0.821) after training, but this did not reach statistical significance (*P* = 0.052). Even though all AUCs based on PRI‐MUS scoring improved, these changes were not statistically significant. All graphs and results are presented in Fig. 2 and Table S2.

### Management Recommendations

**Fig. 3:**
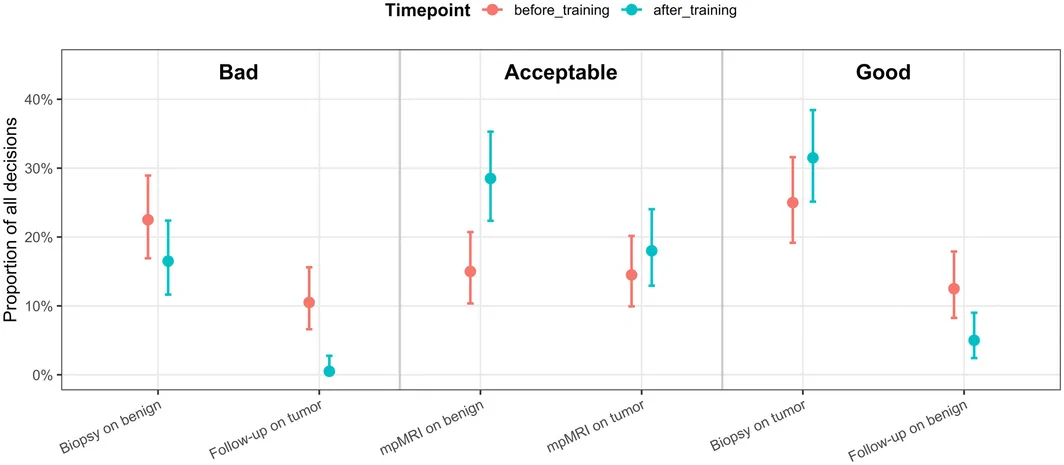
Distribution of management recommendations before and after training. Management recommendations before and after training categorised according to the pre‐defined three‐level decision framework. ‘Good’ decisions included biopsy recommendations in csPCa‐positive cases and follow‐up recommendations in biopsy‐negative cases. ‘Bad’ decisions included biopsy recommendations in biopsy‐negative cases and follow‐up recommendations in csPCa‐positive cases. The mpMRI recommendations were categorised as ‘acceptable’ independent of pathology status. Points represent proportions of all decisions; error bars indicate 95% CIs.

Following training, readers demonstrated a shift in management strategy, with fewer follow‐up recommendations (46 vs 11) and increased use of mpMRI (59 vs 93), while biopsy rates remained stable (95 vs 96). Overall binary decision correctness improved significantly from 67% before to 83% after training (*P* = 0.001). Correctness increased for follow‐up decisions (54% vs 91%, *P* = 0.04) and biopsies (53% vs 66%, *P* = 0.08), while mpMRI recommendations were classified as appropriate within the pre‐defined framework. Using the three‐level quality framework, the proportion of poor‐quality decisions decreased significantly (from 33% to 17%), with a corresponding increase in acceptable decisions (*P* = 0.001). Further details on management decisions are presented in Table 4 and Fig. 3.

**Table 4:** Changes in management decisions before vs after training.

Category	Before training, % (*n/N*)	After training, % (*n/N*)	*P*
Decision distribution			
Follow‐up	23.0 (46/200)	5.5 (11/200)	NA
mpMRI	29.5 (59/200)	46.5 (93/200)	NA
Biopsy	47.5 (95/200)	48.0 (96/200)	NA
Decision correctness			
Overall	67.0 (134/200)	83.0 (166/200)	0.001
Follow‐up	54.3 (25/46)	90.9 (10/11)	0.04
mpMRI	100.0 (59/59)	100.0 (93/93)	1.00
Biopsy	52.6 (50/95)	65.6 (63/96)	0.08
Decision quality			
Bad	33.0 (66/200)	17.0 (34/200)	0.001
Acceptable	29.5 (59/200)	46.5 (93/200)	
Good	37.5 (75/200)	36.5 (73/200)	

Decision distribution describes the proportion of follow‐up, mpMRI, and biopsy recommendations before and after training. Decision correctness was evaluated using the pre‐defined binary framework described in the Methods section. Decision quality was categorised as ‘good’, ‘acceptable’, or ‘poor’ according to the pre‐defined three‐level framework. Binary comparisons were analysed using Fisher's exact tests; overall distribution of decision quality was analysed using a global Fisher 2 × 3 test.

## Discussion

This study showed that a structured 2‐day training programme improved cohort‐level performance in the assessment of microUS images for csPCa in a controlled setting. Training improved sensitivity and overall accuracy, indicating better identification of clinically significant disease. While the three‐tiered subjective risk score of 1–10 showed a statistically significant improvement in AUC, PRI‐MUS scoring remained challenging to apply effectively in this non‐representative cohort. This suggests that structured categorical scoring systems may require greater experience and repeated case exposure than broader subjective pattern recognition.

Importantly, mixed‐effects modelling demonstrated that the training effect was reproducible across the two independent training sessions. Baseline performance did not differ between cohorts, and the magnitude of improvement after training was comparable, suggesting the observed effects were attributable to the curriculum rather than cohort‐specific differences.

Clinical decision‐making after microUS remains an important area of investigation, particularly as randomised trials evaluating biopsy‐avoidance strategies are ongoing. Although biopsy rates remained stable before and after training, the proportion of biopsied patients with csPCa increased from 52.6% to 65.6%. Following training, participants became more cautious in assigning patients to PSA follow‐up, with only one inappropriate follow‐up recommendation in a case with csPCa. When unnecessary biopsies and inappropriate follow‐up assignments were considered together, the rate of false decisions decreased from 33% to 17%. While these simplified recommendations do not fully reflect real‐world clinical decision‐making, where PSA kinetics, MRI findings, and other patient‐specific factors influence subsequent diagnostic procedures, they suggest increased confidence and more appropriate management within the controlled framework of this study.

Despite recent publications demonstrating non‐inferiority of microUS to mpMRI in a biopsy‐all strategy, adoption of microUS as an imaging modality remains limited. The overall guidance on its use remains heavily influenced by the producer of the US system. This starkly contrasts with the current status of mpMRI for PCa detection. While PRI‐MUS currently remains a pure imaging interpretation system, PI‐RADS has become a system for the acquisition, interpretation and reporting of mpMRI [17]. Furthermore, consensus conferences such as that organised through the European Society of Urogenital Radiology (ESUR) and the European Association of Urology Section of Urological Imaging (ESUI) provide guidance for the education of radiologists learning prostate mpMRI for cancer detection [15]. The mpMRI training recommendations emphasise structured education with supervised reporting and defined experience thresholds, including interpretation of at least 100 supervised cases. Together with the PI‐RADS and PI‐QUAL frameworks, these initiatives have improved consistency in mpMRI interpretation.

To ensure widespread high‐quality imaging with microUS, a similar process as that of mpMRI will be necessary. Enabling high‐quality education in microUS interpretation and providing a supervised format for novices before they start independent reporting was the goal of this training study. While the participants of this study have likely not reached expert level, significant improvements in scoring and decision‐making were seen after the completion of the 85 example cases included in the training. While multiple studies examining mpMRI learning curves exist, the only similar study in microUS to date examines a structured training programme by Cash et al. [18]. The four‐stage training programme includes six on‐line learning modules and 1 h of didactic instruction; trainees then undergo their first 10–15 live cases under supervision, followed by case reviews after 10, 20, 40, and 90 cases. The study showed statistically significant improvement in the detection of csPCa from 40% to 57%. Specificity increased from 16% to 37% after 90 cases. However, the examinations included patients who had previously undergone mpMRI, with results available to the participants, which could have biased the results. Under these considerations, with nearly equal specificity and NPV, the results of the examined curriculum appear highly promising. By enabling structured image‐based training before independent clinical use, the curriculum offers a scalable and less time‐intensive alternative to prolonged one‐to‐one proctoring during the early phase of learning microUS.

Among the findings, the study revealed that the subjective overall risk score, taught using a structured three‐tier system (1–3 ‘likely benign’, 4–5 ‘equivocal’, ≥6 ‘suspicious’), demonstrated a more pronounced improvement than PRI‐MUS scoring. AUC values for both the overall risk score and the peripheral‐zone score increased significantly from 0.531 to 0.701 and from 0.507 to 0.672, respectively. While the AUC of PRI‐MUS scoring also improved, changes were weaker and not statistically significant. PRI‐MUS scoring was taught using curated examples closely aligned with the original definitions by Ghai et al. [20]. The stronger improvement in subjective risk scoring suggests that readers may first develop a broader visual impression of malignant vs benign appearances before they are able to reliably translate these findings into criteria‐based scoring systems such as PRI‐MUS. Nonetheless, repeated exposure to PRI‐MUS scoring with targeted feedback may help improve consistency in lesion classification. This will be an important focus for future refinement of the curriculum.

Our group previously proposed integrating transitional‐zone risk scoring into the PRI‐MUS framework [17]. Although not statistically significant due to the small number of transitional‐zone tumours, the observed AUC increase suggests that dedicated training may improve detection of this challenging group of tumours with less reliable definitions of high‐risk features.

### Limitations

This study has several methodological aspects that should be considered when interpreting the findings. First, the evaluation design did not include repeated assessment of the same cases by the same reader. Instead, different case subsets were evaluated before and after training, such that analyses reflected pooled cohort‐level performance rather than paired within‐reader changes. However, this was a design choice made to avoid a test–retest bias and while case‐mix differences might have played a role for the individual raters, as all test‐dataset cases were assessed only once per timepoint in each training run, on a cohort level the same cases were assessed before and after training.

The study cohort was relatively small and heterogeneous, which limits conclusions for any specific professional groups. Although the exploratory interaction model did not show a significant difference in post‐training effect by professional background, the inclusion of three medical students could limit generalisability to routine clinical microUS users. In addition, the test set was enriched for patients with PCa and consisted exclusively of men already selected for biopsy via previous mpMRI. Furthermore, examinations with very poor image quality were excluded. This resulted in a higher prevalence of csPCa and a more controlled imaging dataset than would be expected in routine clinical practice, potentially leading to over‐ or underestimation of diagnostic performance in lower‐prevalence settings. Finally, the study focused on blinded interpretation of pre‐acquired images and did not account for real‐time image acquisition or biopsy performance.

As a limitation to this study, however also possibly to microUS in general, specificity remained low and did not significantly improve. This finding should be interpreted in the context of the study population, which consisted exclusively of patients already selected for biopsy, most commonly because of suspicious mpMRI findings. Consequently, a low specificity would be expected even with accurate image interpretation. Nevertheless, the persistently low specificity after training also suggests that distinguishing benign from suspicious microUS findings remains challenging during early training and may require further experience.

A strength of the study is the use of a fixed, biopsy‐validated test dataset independent of the training cases, allowing a controlled comparison across timepoints while minimising bias related to prior case exposure.

## Conclusions

The Mannheim Micro‐Ultrasound Curriculum improved microUS interpretation and management decisions among inexperienced readers and reduced inappropriate management recommendations after training. As clinical adoption of microUS continues, dedicated training and quality assurance frameworks will be essential for consistent clinical implementation.

## Author Contributions

**Maximilian Philip Wessendorf:** conceptualisation, methodology, investigation, data curation, formal analysis, writing – original draft, writing – review and editing. **Maurin Helen Mangold:** investigation, writing – review and editing. **Gloria Baumann:** investigation. **Nektarios Winter:** data curation. **Johannes Jannik Braun:** investigation. **Jiri Lehmberg:** data curation. **Alexander Studier‐Fischer:** resources. **Christoph Brochhausen:** resources. **Maurice Stephan Michel:** resources. **Sigrid V. Carlsson:** investigation. **Karl‐Friedrich Kowalewski:** writing – review and editing, resources. **Caelán Max Haney‐Aubert:** conceptualisation, methodology, investigation, writing – review and editing, supervision, project administration, resources.

## Disclosure of Interests

The authors declare that they have no financial or non‐financial conflicts of interest related to this work. The study was conducted using the ExactVu™ system (Exact Imaging Inc.); no funding or support was received from the manufacturer.

## Declaration of Artificial Intelligence (AI) Usage

During the preparation of this work, the authors used Chat Generative Pre‐Trained Transformer 5 (ChatGPT‐5; OpenAI, Inc., San Francisco, CA, USA) for language and spelling review. No content‐related analysis, data processing, or literature research was conducted using this or any other AI tool. After using this service, the authors carefully reviewed and edited the text as needed and take full responsibility for the content of the published article.

## Funding

This research was supported by a grant from the Dietmar Hopp Foundation. The funder had no role in the study design, data collection, data analysis, data interpretation, manuscript preparation, or decision to publish.

## Ethics Approval

Institutional ethics committee approval was obtained for the microUS database (2023–645‐AF 5) and the reader study (2024–653) at Mannheim University Medical Centre.

## Study Registration

The study was registered in the German Clinical Trials Register (DRKS00039253).

References1

Sung
H
, 
Ferlay
J
, 
Siegel
RL
 et al. Global cancer statistics 2020: GLOBOCAN estimates of incidence and mortality worldwide for 36 cancers in 185 countries. CA Cancer J Clin
2021; 71: 209–249
33538338
10.3322/caac.216602

Cornford
P
, 
van den Bergh
RCN
, 
Briers
E
 et al. EAU‐EANM‐ESTRO‐ESUR‐ISUP‐SIOG guidelines on prostate cancer—2024 update. Part I: screening, diagnosis, and local treatment with curative intent. Eur Urol
2024; 86: 148–163
38614820
10.1016/j.eururo.2024.03.0273

Roobol
MJ
, 
de Vos
II
, 
Månsson
M
 et al. European study of prostate cancer screening—23‐year follow‐up. N Engl J Med
2025; 393: 1669–1680
41160819
10.1056/NEJMoa25032234

Wei
JT
, 
Barocas
D
, 
Carlsson
S
 et al. Early detection of prostate cancer: AUA/SUO guideline part I—prostate cancer screening. J Urol
2023; 210: 46–53
37096582
10.1097/JU.0000000000003491PMC110607505

Ahmed
HU
, 
El‐Shater Bosaily
A
, 
Brown
LC
 et al. Diagnostic accuracy of multi‐parametric MRI and TRUS biopsy in prostate cancer (PROMIS): a paired validating confirmatory study. Lancet
2017; 389: 815–822
28110982
10.1016/S0140-6736(16)32401-16

Haider
MA
, 
Brown
J
, 
Yao
X
 et al. Multiparametric magnetic resonance imaging in the diagnosis of clinically significant prostate cancer: an updated systematic review. Clin Oncol (R Coll Radiol)
2021; 33: e599–e612
34400038
10.1016/j.clon.2021.07.0167

Murali
S
, 
Ding
H
, 
Adedeji
F
 et al. Bringing MRI to low‐ and middle‐income countries: directions, challenges and potential solutions. NMR Biomed
2024; 37: e4992
37401341
10.1002/nbm.49928

El Khoury
CJ
, 
Freedland
SJ
, 
Gandhi
K
 et al. Disparities in the utilization of magnetic resonance imaging for prostate cancer detection: a population‐based study. J Natl Cancer Inst
2025; 117: 270–278
39312683
10.1093/jnci/djae234PMC118074369

Vergara
TV
, 
Magsanoc
JM
, 
Mendoza
MJ
 et al. Overcoming barriers to prostate cancer care in The Philippines. Front Urol
2024; 3: 1336179
40778027
10.3389/fruro.2023.1336179PMC1232734710

Kinnaird
A
, 
Luger
F
, 
Cash
H
 et al. Microultrasonography‐guided vs MRI‐guided biopsy for prostate cancer diagnosis: the OPTIMUM randomized clinical trial. JAMA
2025; 333: 1679–1687
40121537
10.1001/jama.2025.3579PMC1193142511

Hofbauer
SL
, 
Luger
F
, 
Harland
N
 et al. A non‐inferiority comparative analysis of microultrasonography and MRI‐targeted biopsy in men at risk of prostate cancer. BJU Int
2022; 129: 648–654
34773679
10.1111/bju.1563512

Purysko
AS
, 
Baroni
RH
, 
Giganti
F
 et al. PI‐RADS version 2.1: a critical review from the AJR special series on radiology reporting and data systems. AJR Am J Roentgenol
2021; 216: 20–32
32997518
10.2214/AJR.20.2449513

Giganti
F
, 
Allen
C
, 
Emberton
M
 et al. Prostate imaging quality (PI‐QUAL): a new quality control scoring system for multiparametric MRI of the prostate from the PRECISION trial. Eur Urol Oncol
2020; 3: 615–619
32646850
10.1016/j.euo.2020.06.00714

de Rooij
M
, 
Allen
C
, 
Twilt
JJ
 et al. PI‐QUAL version 2: an update of a standardised scoring system for the assessment of image quality of prostate MRI. Eur Radiol
2024; 34: 7068–7079
38787428
10.1007/s00330-024-10795-4PMC1151915515

de Rooij
M
, 
Israël
B
, 
Tummers
M
 et al. ESUR/ESUI consensus statements on multiparametric MRI for the detection of clinically significant prostate cancer: quality requirements for image acquisition, interpretation and radiologists' training. Eur Radiol
2020; 30: 5404–5416
32424596
10.1007/s00330-020-06929-zPMC747699716

DuBois
J
, 
Smani
S
, 
Golos
A
, 
Rivera Lopez
C
, 
Lokeshwar
SD
. Micro‐ultrasound in the detection of clinically significant prostate cancer: a comprehensive review and comparison with multiparametric MRI. Tomography
2025; 11: 80
40710898
10.3390/tomography11070080PMC1229932417

Haney‐Aubert
CM
, 
Mangold
MH
, 
Kowalewski
KF
. What do we need for PRI‐MUS 2.0?
J Urol
2025; 214: 339–340
40554770
10.1097/JU.000000000000465518

Cash
H
, 
Hofbauer
SL
, 
Shore
N
 et al. Prostate cancer detection by novice micro‐ultrasound users enrolled in a training program. SIUJ
2022; 3: 62–68
19

Lughezzani
G
, 
Maffei
D
, 
Saita
A
 et al. Diagnostic accuracy of microultrasound in patients with a suspicion of prostate cancer at magnetic resonance imaging: a single‐institution prospective study. Eur Urol Focus
2021; 7: 1019–1026
33069624
10.1016/j.euf.2020.09.01320

Ghai
S
, 
Eure
G
, 
Fradet
V
 et al. Assessing cancer risk on novel 29 MHz micro‐ultrasound images of the prostate: creation of the PRI‐MUS protocol. J Urol
2016; 196: 562–569
26791931
10.1016/j.juro.2015.12.093

## Supporting information


**Video S1.** Video abstract of the Mannheim Micro‐Ultrasound Curriculum.

**Fig. S1.** Reproducibility of discrimination improvement.
**Fig. S2.** Distribution of overall subjective risk scores before and after training.
**Table S1.** Mixed‐effects analysis of reproducibility across training sessions.
**Table S2.** The ROC analyses of PRI‐MUS and subjective risk scores before and after training.

## Data Availability

The data that support the findings of this study are available from the corresponding author upon reasonable request. Due to patient privacy regulations, data are not publicly available.
