# Supplementary material for: Structured micro‐ultrasonography training improves prostate cancer detection and management decisions
Source: BJU Int. 2026 Jun 18;138(4):630–9. doi: 10.1111/bju.70360 (PMC13550805; doi:10.1111/bju.70360)
Supplement: Supplementary file 1 [file BJU-138-630-s002.docx]

# Supplementary

**Supplementary Figure 1: Reproducibility of discrimination improvement**

*Area under the curve (AUC) before and after training for the overall risk score and PRI-MUS overall score across the two independent training sessions. Error bars represent DeLong 95% confidence intervals.*

**Supplementary Table 1: Mixed-effects analysis of reproducibility across training sessions**

| Effect | Odds Ratio (95% CI) | p-value |
| --- | --- | --- |
| **Baseline (Session 1 before training)** | 0.96 (0.63–1.46) | 0.85 |
| **Training effect (Session 1)** | 2.13 (1.20–3.79) | 0.01 |
| **Baseline difference (Session 2 vs Session 1)** | 1.13 (0.65–1.97) | 0.67 |
| **Training effect difference (Session 2 vs Session 1)** | 0.71 (0.32–1.59) | 0.41 |

Mixed-effects logistic regression model assessing reproducibility of the training effect across the two independent training sessions. Random intercepts were included for reader and case. Session 1 before training served as the reference category. CI = confidence interval.

**Supplementary Table 2: ROC analyses of PRI-MUS and subjective risk scores before and after training**

| Model / score | Before training | After training | P-Value (DeLong) |
| --- | --- | --- | --- |
| **Risk score (overall)** | 0.531 (0.451–0.611) | 0.701 (0.631–0.771) | 0.002 |
| **Risk score (peripheral zone)** | 0.507 (0.424–0.590) | 0.672 (0.597–0.746) | 0.004 |
| **Risk score (anterior)** | 0.523 (0.395–0.651) | 0.700 (0.578–0.821) | 0.052 |
| **PRI-MUS (overall)** | 0.519 (0.441–0.596) | 0.610 (0.538–0.681) | 0.09 |
| **PRI-MUS (peripheral)** | 0.524 (0.444–0.604) | 0.589 (0.513–0.666) | 0.25 |
| **PRI-MUS (anterior)** | 0.484 (0.368–0.599) | 0.494 (0.353–0.634) | 0.91 |

AUCs are presented with DeLong 95% confidence intervals. P-values were calculated using DeLong tests comparing ROC curves before and after training. AUC = area under the receiver operating characteristic curve; ROC = receiver operating characteristic; PRI-MUS = Prostate Risk Identification using Micro-Ultrasound; CI = confidence interval.

**Supplementary Figure 2: Distribution of overall subjective risk scores before and after training**

*Density distribution of overall subjective risk scores assigned to csPCa-positive and biopsy-negative cases before and after training. Dashed lines indicate mean risk scores for each group.*
